# Prognostic significance of gastrointestinal dysfunction in critically ill patients with COVID-19

**DOI:** 10.62675/2965-2774.20240020-en

**Published:** 2024-10-21

**Authors:** Ricardo Antônio Correia Lima, Annika Reintam Blaser, Júlia Falconiere Paredes Ramalho, Barbara Cristina de Almeida Campos Lacerda, Gabriela Sadigurschi, Paula Fonseca Aarestrup, Rafael Aguilar Sales, João Mansur, Roberto Muniz Ferreira

**Affiliations:** 1 Intensive Care Unit Hospital Samaritano Rio de Janeiro RJ Brazil Intensive Care Unit, Hospital Samaritano - Rio de Janeiro (RJ), Brazil.; 2 University of Tartu Department of Anaesthesiology and Intensive Care Tartu Estonia Department of Anaesthesiology and Intensive Care, University of Tartu - Tartu, Estonia.; 3 Hospital Samaritano Department of Cardiology Rio de Janeiro RJ Brazil Department of Cardiology, Hospital Samaritano - Botafogo, Rio de Janeiro (RJ), Brazil.

**Keywords:** COVID-19, Coronavirus infections, Gastrointestinal motility, Gastroparesis, Critical care, Prognosis, Hospital mortality, Hospitalization, Intensive care units

## Abstract

**Objective:**

To analyze in-hospital and 1-year morbidity and mortality associated with acute gastrointestinal dysfunction in critically ill patients with COVID-19 via a prespecified scoring system.

**Methods:**

Between March and July 2020, consecutive hospitalized patients with COVID-19 from a single institution were retrospectively analyzed by medical chart review. Only those who remained in the intensive care unit for more than 24 hours were included. Gastrointestinal dysfunction was assessed according to a predefined 5-point progressive gastrointestinal injury scoring system, considering the first 7 days of hospitalization. Laboratory data, comorbidities, the need for mechanical ventilation, the duration of intensive care unit stay, and subsequent in-hospital and 1-year mortality rates were also recorded.

**Results:**

Among 230 patients who were screened, 215 were included in the analysis. The median age was 68 years (54 - 82), and 57.7% were male. The total gastrointestinal dysfunction scores were 0 (79.1%), I (15.3%), II (4.7%), III (0.9%), and IV (0%). Any manifestation of gastrointestinal dysfunction was present in 20.9% of all patients and was associated with longer lengths of stay (20 days [11 - 33] *versus* 7 days [4 – 16]; p < 0.001] and higher C-reactive protein levels on admission (12.8mg/mL [6.4 - 18.4] *versus* 5.7mg/mL [3.2 - 13.4]; p < 0.001). The gastrointestinal dysfunction score was significantly associated with mortality (OR 2.8; 95%CI 1.7 - 4.8; p < 0.001) and the need for mechanical ventilation (OR 2.8; 95%CI 1.7 - 4.6; p < 0.001). Both in-hospital and 1-year death rates progressively increased as gastrointestinal dysfunction scores increased.

**Conclusion:**

In the current series of intensive care unit patients with COVID-19, gastrointestinal dysfunction severity, as defined by a prespecified scoring system, was predictive of adverse in-hospital and 1-year outcomes.

## INTRODUCTION

Gastrointestinal dysfunction (GID) occurs frequently in critically ill patients and is associated with adverse outcomes. Sixty-two percent of patients admitted to intensive care units (ICUs) may exhibit one or more gastrointestinal manifestations for at least 24 hours. However, diagnosis of GID in the ICU is complex and has historically relied mostly on clinical symptoms.^(1,2)^ With the aim of rating GID under these conditions, the acute gastrointestinal injury (AGI) grade classification was developed to assess gastrointestinal tract function in critically ill patients. Since this classification was developed descriptively, its application in clinical studies has been limited due to high interobserver variability.^(3)^

In this context, the GID score was created to provide a clear evaluation method that is readily available at the bedside, with minimal subjectivity and increased reproducibility. The GID grading system is fundamentally based on the AGI score but replaces the overall clinical impression of the AGI classification with detailed gastrointestinal symptoms, making it a tool for potentially predicting outcomes in hospitalized patients.^(3)^ This study aimed to analyze the in-hospital and 1-year morbidity and mortality associated with acute gastrointestinal dysfunction in critically ill patients with coronavirus disease 2019 (COVID-19) using a predefined scoring system.

## METHODS

In this single-center observational study, a consecutive sample comprising all patients with symptomatic COVID-19 admitted between March 12 and July 8, 2020, was retrospectively analyzed via medical chart review to determine the prevalence of GID. Among 230 patients who were screened and had a positive real-time reverse transcription–polymerase chain reaction (RT-PCR) test for severe acute respiratory syndrome coronavirus 2 (SARS-CoV-2), 215 remained in the ICU for more than 24 hours and were included in the analysis.

Gastrointestinal dysfunction was assessed via a GID score previously developed by Reintam Blaser et al.^(4)^ Patients were classified according to a 5-point progressive gastrointestinal injury scoring system: GID 0 (no risk); GID 1 (high risk); GID 2 (gastrointestinal tract dysfunction); GID 3 (gastrointestinal tract failure); and GID 4 (life-threatening dysfunction). The definitions of each level of GID are presented in table 1.

**Table 1:** Gastrointestinal dysfunction score definitions

GID score	Definition
0	No symptoms or only 1 of the following factors with normal oral intake: absence of intestinal peristalsis, vomiting, gastric residual volume > 200mL, gastrointestinal paralysis or adynamic ileus, abdominal distension, nonsevere diarrhea, gastrointestinal bleeding without transfusion.
1	2 of the following: no oral intake, absence of intestinal peristalsis, vomiting, gastric residual volume > 200mL, gastrointestinal paralysis or adynamic ileus, abdominal distension, nonsevere diarrhea, gastrointestinal bleeding without transfusion.
2	≥ 3 symptoms of GID 1 or up to 2 of the following factors: severe diarrhea, gastrointestinal bleeding requiring transfusion.
3	≥ 3 of the following factors: prokinetic use, gastrointestinal paralysis or adynamic ileus, abdominal distension, severe diarrhea, gastrointestinal bleeding requiring transfusion.
4	1 of the following factors: gastrointestinal bleeding leading to hemorrhagic shock or mesenteric ischemia.

GID - gastrointestinal dysfunction.

Source: Adapted from Reintam Blaser A, Padar M, Mändul M, Elke G, Engel C, Fischer K, et al. Development of the gastrointestinal dysfunction score (GIDS) for critically ill patients - A prospective multicenter observational study (iSOFA study). Clin Nutr. 2021;40(8):4932-40.

Strict criteria were considered for every gastrointestinal clinical manifestation. Vomiting was characterized as the occurrence of any visible regurgitation of gastric content regardless of quantity. Diarrhea was defined as ≥ 3 episodes of loose or liquid stools per day, with a total volume > 250mL, while severe diarrhea was characterized ≥ 5 episodes or a total volume ≥ 1000mL. Gastrointestinal bleeding was identified by the presence of visible blood in vomit, gastric aspirate, or stool. Adynamic ileus was defined as the absence of stool for ≥ 3 consecutive days, and intestinal obstruction was considered when its diagnosis was documented in the medical chart. These variables were recorded during the first 7 days of ICU admission and were collected by 4 previously trained physicians. In addition to the GID score, laboratory data, the presence of comorbidities, the use of invasive mechanical ventilation, the duration of ICU stay, and subsequent in-hospital and 1-year mortality rates were also documented. Survival after hospital discharge was determined by consulting a public online database of births and deaths managed by the regional judiciary system, and follow-up data available for all included patients for at least 1 year.

Stata^®^ version 11.0 software was used for statistical analysis. Categorical variables were analyzed with Pearson's χ2 test, the Kruskal-Wallis test, and Fisher's exact test. Continuous variables are expressed as the median and interquartile range (25th to 75th percentile) and were further evaluated via the Wilcoxon-Mann-Whitney test. Clinical variables with known prognostic value and those found significant in the univariate analysis were included in a multivariate logistic regression model to identify independent predictors of in-hospital and 1-year outcomes. A p value < 0.05 was considered statistically significant.

The study conforms to the guidelines of the Declaration of Helsinki and received appropriate institutional review board approval on September 16^th^, 2022, under project number 5.647.896. Informed consent was not required due to the retrospective nature of the study.

## RESULTS

Among 230 patients who were screened, 215 remained in the ICU for more than 24 hours and were included in the analysis. The median age of the hospitalized patients was 68 years (54 - 82) and the majority (57.7%) were male. The complete clinical and laboratory baseline characteristics are listed in table 2. The total duration of hospitalization was 9 days (5 - 21) and 21.4% of patients required mechanical ventilation. The in-hospital mortality rate was 15.8% (34 deaths), with an additional 8 deaths recorded after 1 year, resulting in a total mortality rate of 19.3%.

**Table 2:** Clinical and laboratory baseline characteristics of hospitalized patients in the intensive care unit

Variable	Total (n = 215)	GID score = 0 (n = 170)	GID score ≥ 1 (n = 45)	p value
Age (years)	68 (54 - 82)	67 (52 - 79)	71 (61 - 88)	0.002*
Male	124 (57.7)	101 (59.4)	23 (51.1)	0.32
BMI (kg/m^2^)	26.8 (23.9 - 30.1)	26.2 (23.8 - 29.5)	29 (25 - 31.6)	0.08
Hypertension	113 (52.6)	89 (52.4)	24 (53.3)	0.91
Diabetes	60 (27.9)	47 (27.7)	13 (28.9)	0.87
Cardiovascular disease	25 (11.6)	16 (9.4)	9 (20)	0.049*
Oxygen saturation %	93 (91 - 95)	94 (91 - 96)	92 (88 - 94)	< 0.001*
Noninvasive ventilation	78 (36.3)	47 (27.7)	31 (68.9)	< 0.001*
Hemoglobin (mg/dL)	13.4 (12 - 14.6)	13.4 (12.4 - 14.6)	13 (11.1 - 14.4)	0.14
Leukocyte count (cells/mm^3^)	5920 (4,590 - 8,300)	5755 (4,330 - 7,860)	7660 (5,350 - 10,480)	< 0.001*
C-reactive protein (mg/mL)	6.7 (3.3 - 14.1)	5.7 (3.2 - 13.4)	12.8 (6.4 - 18.4)	< 0.001*
D-dimer (ng/mL)	795 (465 - 1,446)	803 (459 - 1,446)	660 (473 - 1,366)	0.78

GID - gastrointestinal dysfunction; BMI - body mass index.

* p values < 0.05. Values are expressed as medians (interquartile ranges) or n (%).

The total GID scores were as follows: 0 (79.1%), I (15.3%), II (4.7%), III (0.9%), and IV (0%). Any manifestation of gastrointestinal dysfunction (GID ≥ 1) was present in 20.9% of all patients, with decreased bowel motility being the most common finding in this subgroup (53.3%), followed by diarrhea (31.1%). There were no cases of mesenteric ischemia, intestinal obstruction, or compartment syndrome. Prokinetic medications were prescribed to 34.9% of all patients and 57.8% of those with GID. Patients with GID scores ≥ 1 had higher C-reactive protein (CRP) levels at admission (12.8mg/mL [6.4 - 18.4], *versus* 5.7mg/mL [3.2 - 13.4]; p < 0.001), longer lengths of stay (20 days [11-33], *versus* 7 days [4 – 16]; p < 0.001), greater mechanical ventilation requirements (51.1% *versus* 13.5%; p < 0.001), and higher mortality during hospitalization (33.3% *versus* 11.2%; p < 0.001).

**Figure 1:**
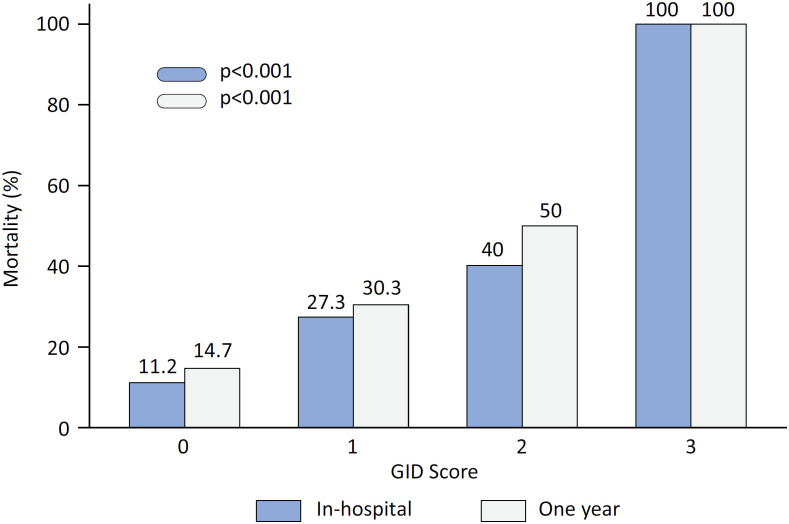
In-hospital and 1-year mortality according to gastrointestinal dysfunction scores determined in the first 7 days of intensive care unit admission.

According to the univariate analysis, the overall GID score was a significant predictor of in-hospital mortality (odds ratio [OR] 2.8; 95% confidence interval [95%CI] 1.7 - 4.8; p < 0.001) and the need for mechanical ventilation (OR 2.8; 95%CI 1.7 - 4.6; p < 0.001). Both in-hospital and 1-year death rates progressively increased as GID score increased (Figure 1). The multivariate logistic model for in-hospital and 1-year outcomes included age, previous coronary artery disease, GID score, C-reactive protein level, leukocyte count, systolic blood pressure, and oxygen saturation at admission. Even after adjustment, GID remained significantly associated with the composite outcome of in-hospital death or mechanical ventilation and subsequent 1-year mortality (Tables 3 and 4).

GID - gastrointestinal dysfunction.

**Table 3:** Predictors of in-hospital death or mechanical ventilation

Variable	Univariate analysis OR (95%CI)	p value	Multivariate analysis OR (95%CI)	p value
Age	1.1 (1.0 - 1.1)	< 0.001*	1.0 (1.0 - 1.1)	0.002*
Coronary artery disease	1.7 (0.7 - 4.1)	0.23	0.8 (0.2 - 2.7)	0.73
GID Score	4.1 (2.3 - 7.2)	< 0.001*	3.3 (1.7 - 6.4)	< 0.001*
Oxygen saturation	0.9 (0.8 - 0.9)	0.002*	0.9 (0.8 - 1.0)	0.15
Systolic blood pressure	0.9 (0.9 - 1.0)	0.009*	0.9 (0.9 - 1.0)	0.009*
C-reactive protein	1.1 (1.1 - 1.2)	< 0.001*	1.1 (1.0 - 1.1)	0.014*
Leukocyte count	1.1 (1.0 - 1.1)	0.003*	1.0 (0.9 - 1.0)	0.87

OR - odds ratio; 95CI - 95% confidence interval; GID - gastrointestinal dysfunction.

* p values < 0.05.

**Table 4:** Predictors of death 1 year after hospitalization

Variable	Univariate analysis OR (95%CI)	p value	Multivariate analysis OR (95%CI)	p value
Age	1.1 (1.1 - 1.2)	< 0.001*	1.1 (1.1 - 1.2)	< 0.001*
Coronary artery disease	2.1 (0.9 - 5.4)	0.1	0.7 (0.2 - 2.1)	0.48
GID Score	2.7 (1.6 - 4.5)	< 0.001*	2.0 (1.1 - 4.0)	0.048*
Oxygen saturation	0.9 (0.8 - 0.9)	0.01*	0.9 (0.9 - 1.0)	0.08
Systolic blood pressure	0.9 (0.97 - 1.0)	0.1	0.9 (0.9 - 1.0)	0.14
C-reactive protein	1.1 (1.0 - 1.1)	0.03*	1.0 (0.9 - 1.1)	0.89

OR - odds ratio; 95CI - 95% confidence interval; GID - gastrointestinal dysfunction.

* p values < 0.05.

## DISCUSSION

Several forms of digestive manifestations, both severe and non-severe, have been described among those with COVID-19. Data from over 18,000 patients with various clinical presentations from the early stages of the pandemic revealed that more than 30% of individuals reported gastrointestinal symptoms. Diarrhea was the most common manifestation, followed by nausea and vomiting, affecting 11.5% and 6.3% of patients, respectively.^(5)^

In critically ill patients hospitalized for COVID-19, higher rates of gastrointestinal complications, including mesenteric ischemia, bleeding, Ogilvie syndrome, and severe ileus, have also been reported. Even compared to other ICU patients with different illnesses, those with COVID-19 are more likely to experience gastrointestinal complications.^(6)^ However, in other profiles of critically ill patients, these manifestations can largely be explained by a combination of adverse pharmacological events and metabolic disturbances. In the context of COVID-19, however, other factors are also involved. A greater expression of angiotensin-converting enzyme 2 receptors along the intestinal epithelial lining has been hypothesized as a possible explanation, as these receptors may facilitate the interaction between host cells and the SARS-CoV-2 virus. Additionally, virus-induced small vessel thrombosis and enteroneuropathy are other likely triggers of GID that warrant further investigation.^(7)^

Most importantly, such gastrointestinal compromise extends beyond the manifestation of isolated and self-limited symptoms and has been associated with adverse clinical outcomes among COVID-19 patients. A study conducted by Sun et al. aimed to investigate the outcomes of AGI in critically ill patients with COVID-19 and revealed that those with higher AGI grades also had worse clinical variables and subsequently greater in-hospital mortality.^(8)^ These results align with the findings of the present study, as higher GID scores were independently associated with the composite outcome of death or the need for mechanical ventilation. The variables included in the multivariate model represented components of the pathophysiological triad characterizing the acute phase of severe COVID-19: hemodynamic compromise (systolic blood pressure), respiratory failure (oxygen saturation), and systemic inflammation (CRP). These findings further highlight the potential independent prognostic value of the GID score.

Our results also suggested that GID during hospitalization may be an independent predictor of mortality up to 1 year after hospitalization. In addition, various long-term gastrointestinal sequelae could arise in the postacute phase of the disease. Xu et al. evaluated over 150,000 patients who survived beyond the first 30 days of the disease and reported an increased risk of several adverse gastrointestinal outcomes in the first year of follow-up. These included motility disorders, acid-related conditions, functional intestinal disturbances, acute pancreatitis, and hepatobiliary disease. Notably, the risk of such complications was amplified among those with greater disease severity in the acute phase and was apparently minimized by prior vaccination.^(9)^

This study has limitations that must be acknowledged. Data were retrospectively collected from a single center and may not reflect the same results as those from other institutions. Additionally, patients were managed before evidence-based treatments and vaccines were available. The influence of currently recommended therapies for patients requiring hospitalization on gastrointestinal outcomes has been variable, with the combination of dexamethasone and remdesivir possibly having a protective effect.^(10)^ Prognostic scoring systems applied to critically ill patients, such as the Acute Physiology and Chronic Health Evaluation II (APACHE II) or Simplified Acute Physiology Score II (SAPS II), were not used due to limited data, and the results were not adjusted for such clinical characterization.^(11)^ Nevertheless, the variables included in the multivariate models are widely recognized as predictors of clinical severity in the context of COVID-19 and may have mitigated this limitation.^(12)^ Finally, the number of events was low, which restricted a thorough statistical analysis of the results.

## CONCLUSION

In the current series of hospitalized patients with severe COVID-19, the gastrointestinal system was frequently affected. Although symptoms were mostly mild, any manifestation of gastrointestinal dysfunction was predictive of both adverse in-hospital outcomes and 1-year mortality. Future studies should validate these findings by further implementing the gastrointestinal dysfunction score among patients with COVID-19.
