# Supplementary material for: Prognostic significance of gastrointestinal dysfunction in critically ill patients with COVID-19
Source: Crit Care Sci. 2024 Oct 21;36:e20240020en. doi: 10.62675/2965-2774.20240020-en (PMC11554297; doi:10.62675/2965-2774.20240020-en)
Supplement: Translation

# Translation: Importância prognóstica da disfunção gastrintestinal em pacientes graves com COVID-19

**DOI:** 10.62675/2965-2774.20240020-pt

**Authors:** Ricardo Antônio Correia Lima, Annika Reintam Blaser, Júlia Falconiere Paredes Ramalho, Barbara Cristina de Almeida Campos Lacerda, Gabriela Sadigurschi, Paula Fonseca Aarestrup, Rafael Aguilar Sales, João Mansur, Roberto Muniz Ferreira

**Affiliations:** 1 Rio de Janeiro RJ Brasil Unidade de Terapia Intensiva, Hospital Samaritano - Rio de Janeiro (RJ), Brasil.; 2 Tartu Estônia Department of Anaesthesiology and Intensive Care, University of Tartu - Tartu, Estônia.; 3 Botafogo RJ Brasil Departamento de Cardiologia, Hospital Samaritano - Botafogo (RJ), Brasil.

**Keywords:** COVID-19, Infecções por coronavírus, Motilidade gastrointestinal, Gastroparesia, Cuidados críticos, Prognóstico, Mortalidade hospitalar, Hospitalização, Unidades de terapia intensiva

## Abstract

**Objetivo:**

Analisar a morbidade e a mortalidade intra-hospitalar e em 1 ano associadas à disfunção gastrintestinal aguda em pacientes graves com COVID-19 por meio de um sistema de pontuação predefinido.

**Métodos:**

Entre março e julho de 2020, pacientes consecutivos hospitalizados com COVID-19 de uma única instituição foram analisados retrospectivamente por meio de revisão de prontuários médicos. Foram incluídos somente aqueles que permaneceram na unidade de terapia intensiva por mais de 24 horas. A disfunção gastrintestinal foi avaliada segundo um sistema predefinido de pontuação de lesão gastrintestinal progressiva de cinco pontos, considerando os primeiros 7 dias de hospitalização. Também se registraram os dados laboratoriais, as comorbidades, a necessidade de ventilação mecânica, a duração da internação na unidade de terapia intensiva e as taxas de mortalidade intra-hospitalar e em 1 ano.

**Resultados:**

Do total de 230 pacientes examinados, 215 foram incluídos na análise. A idade mediana foi de 68 anos (54 - 82), e 57,7% eram do sexo masculino. Os escores totais de disfunção gastrintestinal foram zero (79,1%), I (15,3%), II (4,7%), III (0,9%) e IV (0%). Qualquer manifestação de disfunção gastrintestinal estava presente em 20,9% de todos os pacientes e foi associada à maior duração da internação (20 dias [11 - 33] *versus* 7 dias [4 - 16]; p < 0,001) e a níveis mais altos de proteína C-reativa na admissão (12,8mg/mL [6,4 - 18,4] *versus* 5,7mg/mL [3,2 - 13,4]; p < 0,001). O escore de disfunção gastrintestinal foi significativamente associado à mortalidade (RC 2,8; IC95% 1,7 - 4,8; p < 0,001) e à necessidade de ventilação mecânica (RC 2,8; IC95% 1,7 - 4,6; p < 0,001). As taxas de mortalidade hospitalar e em 1 ano aumentaram progressivamente com o aumento dos escores de disfunção gastrintestinal.

**Conclusão:**

Na série atual de pacientes de unidades de terapia intensiva com COVID-19, a gravidade da disfunção gastrintestinal, conforme definida por um sistema de pontuação predefinido, foi preditiva de resultados adversos no hospital e em 1 ano.

## INTRODUÇÃO

A disfunção gastrintestinal (DGI) ocorre com frequência em pacientes graves e está associada a desfechos adversos. Dos pacientes admitidos em unidades de terapia intensiva (UTIs), 62% podem apresentar uma ou mais manifestações gastrintestinais por, pelo menos, 24 horas. No entanto, o diagnóstico de DGI na UTI é complexo e, historicamente, tem sido fundamentado principalmente em sintomas clínicos.^(1,2)^ Com o objetivo de classificar a DGI nessas condições, a classificação de grau de lesão gastrintestinal aguda foi desenvolvida para avaliar a função do trato gastrintestinal em pacientes graves. Como essa classificação foi desenvolvida de forma descritiva, sua aplicação em estudos clínicos tem sido limitada devido à alta variabilidade interobservador.^(3)^

Nesse contexto, o escore de DGI foi concebido para fornecer um método de avaliação claro e prontamente disponível à beira do leito, com mínima subjetividade e maior reprodutibilidade. O sistema de classificação de DGI baseia-se fundamentalmente no escore de lesão gastrintestinal aguda, mas substitui a impressão clínica geral da classificação de lesão gastrintestinal aguda por sintomas gastrintestinais detalhados, tornando-a uma ferramenta com potencial de prever desfechos em pacientes hospitalizados.^(3)^ Este estudo objetivou analisar a morbidade e a mortalidade intra-hospitalar e em 1 ano associadas à disfunção gastrintestinal aguda em pacientes graves com a doença pelo coronavírus 2019 (COVID-19) por meio de um sistema de pontuação predefinido.

## MÉTODOS

Neste estudo observacional de centro único, analisou-se retrospectivamente uma amostra consecutiva composta de todos os pacientes com COVID-19 sintomáticos admitidos entre 12 de março e 8 de julho de 2020, por meio de revisão de prontuários médicos, para determinar a prevalência de DGI. Dos 230 pacientes que foram examinados e apresentaram teste positivo de reação em cadeia da polimerase via transcriptase reversa (RT-PCR) do coronavírus da síndrome respiratória aguda grave 2 (SARS-CoV-2), 215 permaneceram na UTI por mais de 24 horas e foram incluídos na análise.

A DGI foi avaliada por meio de um escore de DGI desenvolvido anteriormente por Reintam Blaser et al.^(4)^ Os pacientes foram classificados de acordo com um sistema de escore de DGI progressivo de 5 pontos: DGI zero (sem risco); DGI 1 (alto risco); DGI 2 (disfunção do trato gastrintestinal); DGI 3 (insuficiência do trato gastrintestinal); e DGI 4 (disfunção com risco de vida). As definições de cada nível de DGI são apresentadas na tabela 1.

**Tabela 1:** Definições do escore de disfunção gastrintestinal

Escore de DGI	Definição
0	Sem sintomas ou apenas um dos seguintes fatores com ingestão oral normal: ausência de peristaltismo intestinal, vômito, volume residual gástrico > 200mL, paralisia gastrintestinal ou íleo adinâmico, distensão abdominal, diarreia sem gravidade, sangramento gastrintestinal sem transfusão
1	Duas das seguintes situações: ausência de ingestão oral, ausência de peristaltismo intestinal, vômitos, volume residual gástrico > 200mL, paralisia gastrintestinal ou íleo adinâmico, distensão abdominal, diarreia sem gravidade, sangramento gastrintestinal sem transfusão
2	≥ 3 sintomas de DGI. Um ou até dois dos seguintes fatores: diarreia grave, sangramento gastrintestinal com necessidade de transfusão
3	≥ 3 dos seguintes fatores: uso de procinéticos, paralisia gastrintestinal ou íleo adinâmico, distensão abdominal, diarreia grave, sangramento gastrintestinal que requer transfusão
4	Um dos seguintes fatores: sangramento gastrintestinal ocasionando choque hemorrágico ou isquemia mesentérica

DGI - disfunção gastrintestinal.

Fonte: Adaptado de Reintam Blaser A, Padar M, Mändul M, Elke G, Engel C, Fischer K, et al. Development of the gastrointestinal dysfunction score (GIDS) for critically ill patients - A prospective multicenter observational study (iSOFA study). Clin Nutr. 2021;40(8):4932-40.

Foram considerados critérios rigorosos de cada manifestação clínica gastrintestinal. O vômito foi caracterizado como a ocorrência de qualquer regurgitação visível de conteúdo gástrico, independentemente da quantidade. A diarreia foi definida como ≥ 3 episódios de fezes soltas ou líquidas por dia, com volume total > 250mL, enquanto a diarreia grave foi caracterizada como ≥ 5 episódios ou volume total ≥ 1.000mL. O sangramento gastrintestinal foi identificado pela presença de sangue visível no vômito, no aspirado gástrico ou nas fezes. O íleo adinâmico foi definido como a ausência de fezes por ≥ 3 dias consecutivos, e a obstrução intestinal foi considerada quando seu diagnóstico foi documentado no prontuário médico. Essas variáveis foram registradas durante os primeiros 7 dias de admissão na UTI, tendo sido coletadas por quatro médicos previamente treinados. Além do escore de DGI, também foram documentados dados laboratoriais, presença de comorbidades, o uso de ventilação mecânica invasiva, a duração da internação na UTI e as taxas de mortalidade intra-hospitalar e em 1 ano. A sobrevida após a alta hospitalar foi determinada por meio de consulta a um banco de dados público *on-line* de nascimentos e mortes controlado pelo sistema judiciário regional e por meio de dados de seguimento obtidos de todos os pacientes incluídos por pelo menos 1 ano.

Para a análise estatística, foi utilizado o Stata^®^, versão 11.0. As variáveis categóricas foram analisadas pelo teste do qui-quadrado de Pearson, o teste de Kruskal-Wallis e o teste exato de Fisher. As variáveis contínuas são expressas como mediana e intervalo interquartil (IIQ25 - 75%) e foram avaliadas posteriormente por meio do teste Wilcoxon-Mann-Whitney. As variáveis clínicas com valor prognóstico conhecido e aquelas consideradas significativas na análise univariada foram incluídas em um modelo de regressão logística multivariada para identificar preditores independentes de desfechos intra-hospitalares e em 1 ano. Considerou-se estatisticamente significativo valor de p < 0,05.

O estudo está em conformidade com as diretrizes da Declaração de Helsinque e recebeu a devida aprovação do conselho de revisão institucional em 16 de setembro de 2022, sob o número de projeto 5.647.896. Não foi necessário o consentimento informado devido à natureza retrospectiva do estudo.

## RESULTADOS

Dos 230 pacientes selecionados, 215 permaneceram na UTI por mais de 24 horas e foram incluídos na análise. A idade mediana dos pacientes hospitalizados foi de 68 anos (54 - 82), e a maioria (57,7%) era do sexo masculino. As características clínicas e laboratoriais basais completas estão listadas na tabela 2. A duração total da hospitalização foi de 9 dias (5 - 21), e 21,4% dos pacientes precisaram de ventilação mecânica. A taxa de mortalidade intra-hospitalar foi de 15,8% (34 mortes), com mais 8 mortes registradas após 1 ano, elevando a taxa de mortalidade total para 19,3%.

**Tabela 2:** Características clínicas e laboratoriais basais de pacientes hospitalizados na unidade de terapia intensiva

Variável	Total (n = 215)	Escore DGI = 0 (n = 170)	Escore DGI ≥ 1 (n = 45)	Valor de p
Idade (anos)	68 (54 - 82)	67 (52 - 79)	71 (61 - 88)	0,002*
Sexo masculino	124 (57,7)	101 (59,4)	23 (51,1)	0,32
IMC (kg/m^2^)	26,8 (23,9 - 30,1)	26,2 (23,8 - 29,5)	29 (25 - 31,6)	0,08
Hipertensão	113 (52,6)	89 (52,4)	24 (53,3)	0,91
Diabetes	60 (27,9)	47 (27,7)	13 (28,9)	0,87
Doença cardiovascular	25 (11,6)	16 (9,4)	9 (20)	0,049*
Saturação de oxigênio %	93 (91 - 95)	94 (91 - 96)	92 (88 - 94)	< 0,001*
Ventilação não invasiva	78 (36,3)	47 (27,7)	31 (68,9)	< 0,001*
Hemoglobina (mg/dL)	13,4 (12 - 14,6)	13,4 (12,4 - 14,6)	13 (11,1 - 14,4)	0,14
Contagem de leucócitos (células/mm^3^)	5.920 (4.590 - 8.300)	5.755 (4.330 - 7.860)	7.660 (5.350 - 10.480)	< 0,001*
Proteína C-reativa (mg/mL)	6,7 (3,3 - 14,1)	5,7 (3,2 - 13,4)	12,8 (6,4 - 18,4)	< 0,001*
D-dímero (ng/mL)	795 (465 - 1.446)	803 (459 - 1.446)	660 (473 - 1.366)	0,78

DGI - escore de disfunção gastrintestinal; IMC - índice de massa corporal.

* Valores de p < 0,05. Resultados expressos como medianas (intervalos interquartis) ou n (%).

Os escores de DGI totais foram os seguintes: zero para 79,1%, I para 15,3%, II para 4,7%, III para 0,9% e IV para 0%. Qualquer manifestação de DGI ≥ 1 esteve presente em 20,9% de todos os pacientes, sendo a diminuição da motilidade intestinal o achado mais comum nesse subgrupo (53,3%), seguido de diarreia (31,1%). Não houve casos de isquemia mesentérica, obstrução intestinal ou síndrome compartimental. Prescreveram-se medicamentos procinéticos para 34,9% de todos os pacientes e 57,8% daqueles com DGI. Os pacientes com escores de DGI ≥ 1 apresentaram níveis mais altos de proteína C-reativa (PCR) na admissão (12,8mg/mL [6,4 - 18,4], *versus* 5,7mg/mL [3,2 - 13,4]; p < 0,001), duração mais longa da internação (20 dias [11-33], *versus* 7 dias [4 - 16]; p < 0,001), maior necessidade de ventilação mecânica (51,1% *versus* 13,5%; p < 0,001) e maior mortalidade durante a hospitalização (33,3% *versus* 11,2%; p < 0,001).

De acordo com a análise univariada, o escore de DGI geral foi um preditor significativo de mortalidade hospitalar (razão de chances [RC] 2,8; intervalo de confiança de 95% [IC95%] 1,7 - 4,8; p < 0,001) e da necessidade de ventilação mecânica (RC 2,8; IC95% 1,7 - 4,6; p < 0,001). As taxas de mortalidade hospitalar e em 1 ano aumentaram progressivamente, à medida que o escore de DGI aumentou (Figura 1). O modelo logístico multivariado de desfechos no hospital e em 1 ano incluiu idade, doença arterial coronariana prévia, escore de DGI, teor de proteína C-reativa, contagem de leucócitos, pressão arterial sistólica e saturação de oxigênio na admissão. Mesmo após o ajuste, a DGI permaneceu significativamente associada ao desfecho composto de morte hospitalar ou ventilação mecânica e mortalidade subsequente em 1 ano (Tabelas 3 e 4).

**Figura 1:** Mortalidade intra-hospitalar e em 1 ano segundo a pontuação de disfunção gastrintestinal determinada nos primeiros 7 dias de admissão na unidade de terapia intensiva.

DGI - disfunção gastrintestinal.

**Tabela 3:** Preditores de morte intra-hospitalar ou ventilação mecânica

Variável	Análise univariada RC (IC95%)	Valor de p	Análise multivariada RC (IC95%)	Valor de p
Idade	1,1 (1,0 - 1,1)	< 0,001*	1,0 (1,0 - 1,1)	0,002*
Doença arterial coronariana	1,7 (0,7 - 4,1)	0,23	0,8 (0,2 - 2,7)	0,73
Escore de DGI	4,1 (2,3 - 7,2)	< 0,001*	3,3 (1,7 - 6,4)	< 0,001*
Saturação de oxigênio	0,9 (0,8 - 0,9)	0,002*	0,9 (0,8 - 1,0)	0,15
Pressão arterial sistólica	0,9 (0,9 - 1,0)	0,009*	0,9 (0,9 - 1,0)	0,009*
Proteína C-reativa	1,1 (1,1 - 1,2)	< 0,001*	1,1 (1,0 - 1,1)	0,014*
Contagem de leucócitos	1,1 (1,0 - 1,1)	0,003*	1,0 (0,9 - 1,0)	0,87

RC - razão de chances; IC95% - intervalo de confiança de 95%; DGI - disfunção gastrintestinal.

* Valores de p < 0,05.

**Tabela 4:** Preditores de morte 1 ano após a hospitalização

Variável	Análise univariada RC (IC95%)	Valor de p	Análise multivariada RC (IC95%)	Valor de p
Idade	1,1 (1,1 - 1,2)	< 0,001*	1,1 (1,1 - 1,2)	< 0,001*
Doença arterial coronariana	2,1 (0,9 - 5,4)	0,1	0,7 (0,2 - 2,1)	0,48
Escore de DGI	2,7 (1,6 - 4,5)	< 0,001*	2,0 (1,1 - 4,0)	0,048*
Saturação de oxigênio	0,9 (0,8 - 0,9)	0,01*	0,9 (0,9 - 1,0)	0,08
Pressão arterial sistólica	0,9 (0,97 - 1,0)	0,1	0,9 (0,9 - 1,0)	0,14
Proteína C-reativa	1,1 (1,0 - 1,1)	0,03*	1,0 (0,9 - 1,1)	0,89

RC - razão de chances; IC95% - intervalo de confiança de 95%; DGI - disfunção gastrintestinal.

* Valores de p < 0,05.

## DISCUSSÃO

Várias formas de manifestações digestivas, tanto graves quanto não graves, foram descritas entre as pessoas com COVID-19. Dados de mais de 18 mil pacientes com várias apresentações clínicas dos estágios iniciais da pandemia revelaram que mais de 30% dos indivíduos relataram sintomas gastrintestinais. A diarreia foi a manifestação mais comum, seguida de náuseas e vômitos, afetando 11,5 e 6,3% dos pacientes, respectivamente.^(5)^

Em pacientes graves hospitalizados por COVID-19, também foram relatadas taxas mais altas de complicações gastrintestinais, incluindo isquemia mesentérica, sangramento, síndrome de Ogilvie e íleo grave. Mesmo em comparação com outros pacientes de UTI com diferentes doenças, aqueles com COVID-19 têm maior probabilidade de apresentar complicações gastrintestinais.^(6)^ No entanto, em outros perfis de pacientes graves, essas manifestações podem ser amplamente explicadas por uma combinação de eventos farmacológicos adversos e distúrbios metabólicos. No contexto da COVID-19, no entanto, outros fatores também estão envolvidos. Uma maior expressão de receptores da enzima conversora de angiotensina 2 ao longo do revestimento epitelial intestinal foi considerada uma possível explicação, já que esses receptores podem facilitar a interação entre as células hospedeiras e o SARS-CoV-2. Além disso, a trombose de pequenos vasos induzida pelo vírus e a enteroneuropatia são outros prováveis fatores desencadeantes da DGI que justificam uma investigação mais aprofundada.^(7)^

Mais importante ainda, esse comprometimento gastrintestinal vai além da manifestação de sintomas isolados e autolimitados e tem sido associado a desfechos clínicos adversos entre pacientes com COVID-19. Um estudo conduzido por Sun et al. teve como objetivo investigar os desfechos de lesão gastrintestinal aguda em pacientes graves com COVID-19 e revelou que aqueles com graus mais altos desse tipo de lesão também tinham variáveis clínicas desfavoráveis e, subsequentemente, maior mortalidade hospitalar.^(8)^ Esses resultados se alinham com os achados do presente estudo, pois escores de DGI mais altos foram independentemente associados ao desfecho composto de morte ou necessidade de ventilação mecânica. As variáveis incluídas no modelo multivariado representaram componentes da tríade fisiopatológica que caracteriza a fase aguda da COVID-19 grave: comprometimento hemodinâmico (pressão arterial sistólica), insuficiência respiratória (saturação de oxigênio) e inflamação sistêmica (PCR). Esses achados destacam ainda mais o potencial valor prognóstico independente do escore de DGI.

Nossos resultados também sugeriram que a DGI durante a hospitalização pode ser um preditor independente de mortalidade até 1 ano após a hospitalização. Além disso, várias sequelas gastrintestinais duradouras podem surgir na fase pós-aguda da doença. Xu et al. avaliaram mais de 150 mil pacientes que sobreviveram após os primeiros 30 dias da doença e relataram risco maior de vários desfechos gastrintestinais adversos no primeiro ano de seguimento. Esses desfechos incluíram distúrbios de motilidade, condições relacionadas à acidez, distúrbios intestinais funcionais, pancreatite aguda e doença hepatobiliar. Notavelmente, o risco de tais complicações foi ampliado entre aqueles com maior gravidade da doença na fase aguda e foi aparentemente minimizado pela vacinação prévia.^(9)^

Este estudo tem limitações que devem ser reconhecidas. Os dados foram coletados retrospectivamente em um único centro e podem não refletir os mesmos resultados que os de outras instituições. Além disso, os pacientes foram tratados antes da disponibilidade dos tratamentos baseados em evidências e das vacinas. A influência das terapias atualmente recomendadas para pacientes que necessitam de hospitalização nos desfechos gastrintestinais tem sido variável, sendo que a combinação de dexametasona e remdesivir pode ter um efeito protetor.^(10)^ Os sistemas de pontuação prognóstica aplicados a pacientes graves, como o *Acute Physiology and Chronic Health Evaluation II* (APACHE II) ou o *Simplified Acute Physiology Score* II (SAPS II), não foram utilizados devido à limitação de dados, e os resultados não foram ajustados para essa caracterização clínica.^(11)^ No entanto, as variáveis incluídas nos modelos multivariados são amplamente reconhecidas como preditores de gravidade clínica no contexto da COVID-19 e podem ter atenuado essa limitação.^(12)^ Por fim, o número de eventos foi baixo, o que restringiu uma análise estatística completa dos resultados.

## CONCLUSÃO

Na série atual de pacientes hospitalizados com COVID-19 grave, o sistema gastrintestinal foi frequentemente afetado. Embora os sintomas tenham sido em sua maioria leves, qualquer manifestação de disfunção gastrintestinal foi preditiva de desfechos adversos no hospital e de mortalidade em 1 ano. Estudos futuros devem validar esses achados implementando ainda mais o escore de disfunção gastrintestinal em pacientes com COVID-19.
